# Recent Progress in Liquid Microlenses and Their Arrays for Adaptive and Applied Optical Systems

**DOI:** 10.3390/mi16101158

**Published:** 2025-10-13

**Authors:** Siyu Lu, Zheyuan Cao, Jinzhong Ling, Ying Yuan, Xin Liu, Xiaorui Wang, Jin-Kun Guo

**Affiliations:** School of Optoelectronic Engineering, Xidian University, Xi’an 710071, China

**Keywords:** liquid lens, liquid microlens arrays, adaptive optics, tunable optics, imagings, displays, droplets

## Abstract

Liquid microlenses and their arrays (LMLAs) have emerged as a transformative platform in adaptive optics, offering superior reconfigurability, compactness, and fast response compared to conventional solid-state lenses. This review summarizes recent progress from an application-oriented perspective, focusing on actuation mechanisms, fabrication strategies, and functional performance. Among actuation mechanisms, electric-field-driven approaches are highlighted, including electrowetting for shape tuning and liquid crystal-based refractive-index tuning techniques. The former excels in tuning range and response speed, whereas the latter enables programmable wavefront control with lower optical aberrations but limited efficiency. Notably, double-emulsion configurations, with fast interfacial actuation and inherent structural stability, demonstrate great potential for highly integrated optical components. Fabrication methodologies—including semiconductor-derived processes, additive manufacturing, and dynamic molding—are evaluated, revealing trade-offs among scalability, structural complexity, and cost. Functionally, advances in focal length tuning, field-of-view expansion, depth-of-field extension, and aberration correction have been achieved, though strong coupling among these parameters still constrains system-level performance. Looking forward, innovations in functional materials, hybrid fabrication, and computational imaging are expected to mitigate these constraints. These developments will accelerate applications in microscopy, endoscopy, AR/VR displays, industrial inspection, and machine vision, while paving the way for intelligent photonic systems that integrate adaptive optics with machine learning for real-time control.

## 1. Introduction

LMLAs have emerged as pivotal building blocks in adaptive optics and applied optical systems, representing a transformative pathway for next-generation photonic systems. Unlike conventional solid-state lenses, these fluidic optical components achieve dynamic focal tuning by precisely controlling the deformation of liquid–liquid or liquid–solid interface, or by modulating the refractive index of the medium [1,2,3]. Such mechanisms offer distinctive advantages, including mechanical simplicity, fast response, compact footprint, and low power consumption. Especially, the advancement in array integration has further expanded the degrees of freedom for light-field modulation [4,5]. This lays a solid technological foundation for the development of multifunctional integrated photonic systems, with the potential to transform imaging and display technologies [6,7,8].

Driven by advances in micro/nanofabrication and the emergence of novel functional materials [9], research on LMLAs has made significant progress in device architectures, integration strategies, and application performance. Among various architectures, electric-field-driven designs have emerged as the most integration-friendly solutions, demonstrating remarkable versatility in both static and dynamic optical tasks. From the materials perspective, electrically responsive liquids, ionic liquids, and liquid crystals address stability, efficiency, and programmability challenges, expanding the design space of actuation mechanisms [10,11,12]. From the fabrication perspective, additive manufacturing, semiconductor-derived processing, and dynamic molding offer scalable, customizable, and cost-effective approaches to device realization. The convergence and advancement of novel materials and manufacturing technologies [13,14,15] are accelerating the translation from laboratory prototypes to practical applications in imaging, display, machine vision, and medical endoscopy [16,17,18,19].

This review aims to provide an overview of the recent progress in LMLA technologies, particularly from an application-oriented perspective. It first explores electric-field-driven actuation mechanisms, including shape-tuning approaches (e.g., electrowetting) and refractive-index-tuning (liquid crystal) approaches. The design principles and optimization approaches of liquid microlenses are then briefly discussed, followed by a comparison of the key performance parameters of commonly used liquid materials. Fabrication methodologies—including semiconductor-derived processes, additive manufacturing, and dynamic molding—are critically evaluated in terms of their respective advantages and limitations. Finally, it discusses performance breakthroughs in focal length tuning, field-of-view (FOV) expansion, depth-of-field (DOF) extension, and aberration correction, and it addresses emerging challenges and future opportunities for LMLA integration in next-generation adaptive and applied optical systems.

## 2. Actuation Mechanism

**Figure 1:**
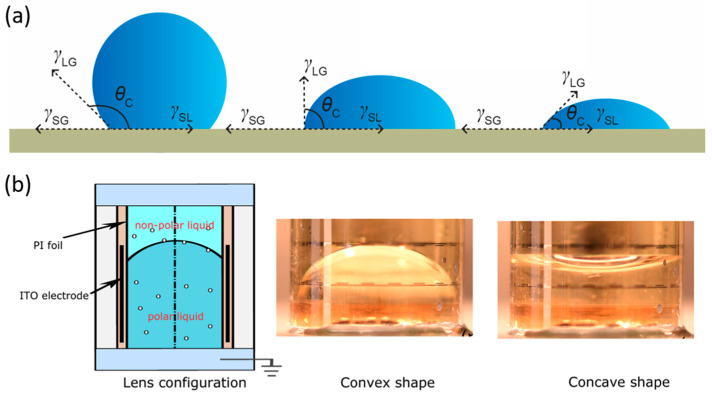
Focusing mechanisms and structures of liquid lenses. (**a**) A droplet resting on a solid surface experiences curvature on its surface influenced by the solid–liquid interfacial tension, represented by the contact angle *θ_c_* [20]. Adapted from Ref. [20] under the terms of the Creative Commons CC BY 4.0 license. (**b**) Electrowetting liquid lens: the meniscus between the two liquids can be switched between convex and concave, enabling convergence and divergence of light [21]. Reprinted with permission from Ref. [21] © Optical Society of America.

Liquid lenses are actuated by external stimuli that either reshape the liquid interface (curvature modulation) or alter the effective refractive index, allowing focal-length tuning. Representative drives include electric, magnetic, acoustic, thermal, and hydraulic methods. Among these, electrowetting is the most mature and widely adopted mechanism (Figure 1a). When a droplet rests on the surface of a solid substrate, its morphology and corresponding contact angle (*θ_c_*) are governed by the balance of interfacial tensions, as described by Young’s equation (Equation (1)). By electrically modifying the interfacial tension at the liquid-solid boundary, the contact angle can be tuned, which in turn controls the interface curvature and the focal length. In practical terms, wettability provides an intuitive description of this behavior: *θ_c_* > 90° indicates hydrophobicity, *θ_c_* < 90° indicates hydrophilicity, and smaller contact angles correspond to stronger wettability, with interfacial tension serving as the underlying physical parameter.

(1) $$\begin{array}{c} \gamma_{LG}\times \cos\theta_{C}=\gamma_{SG}-\gamma_{SL} \end{array}$$

where *γ* denotes the interfacial tension, *θ_c_* represents the contact angle, and subscripts SG, LG, and SL correspond to the solid–gas, liquid–gas, and solid–liquid interfaces, respectively.

Electrowetting liquid lenses operate by controlling the interfacial tension at the liquid-solid interface through the application of a voltage, allowing the focal length of the lens to be adjusted. The variation in the contact angle with voltage is described by the Young–Lippmann equation (Equation (2)):

(2) $$\begin{array}{c} \cos\theta_{C}=\cos\theta_{0}+\frac{\varepsilon }{2\gamma_{LG}d}V^{2} \end{array}$$

In this context, *θ_c_*(*v*) denotes the contact angle under an applied voltage *v*, *θ*_0_ represents the static contact angle, *ε* is the dielectric constant of the insulating layer, *d* is its thickness, and *γ_LG_* refers to the liquid–gas interfacial tension. Under constant temperature and other external conditions, the equation can reliably predict the contact angle variations induced by electrowetting. However, when the applied voltage exceeds a certain threshold, the contact angle no longer decreases and instead reaches a minimum value, a phenomenon known as contact-angle saturation. This saturation hinders complete wetting of the liquid–solid interface, which limits the performance ceiling of the liquid lens. To further enhance the electrowetting effect, a dielectric layer can be introduced between the liquid and the electrode, forming a stable capacitance. The hydrophobic functional layer within the dielectric reduces wettability, enabling the lens to achieve a larger initial contact angle and thus alleviating the limitation of contact-angle saturation and broadening the tunable range of contact angles. In addition, the dielectric layer separates the conductive liquid from the electrode, preventing direct contact that could cause ionization of the conductive liquid. This arrangement not only prevents direct contact between the electrolyte and the electrode, thereby avoiding hydrolysis, but also significantly amplifies the effect of the electric field. The dielectric electrowetting-on-dielectric (EWOD) configuration enables precise control over droplet morphology, providing critical support for the focal length tuning functionality of liquid lenses.

Figure 1b illustrates a typical electrowetting liquid lens configuration. By applying different voltages, the interface can be switched between a convex (converging) and a concave (diverging) shape, enabling bidirectional and reversible tuning of the lens focal length. The lens contains two immiscible transparent liquids: an insulating nonpolar fluid and a non-insulating polar electrolyte aqueous solution. These two liquids possess different refractive indices but similar densities, allowing the liquid–liquid interface to maintain a spherical shape without being affected by gravity. The focal length *f* is quantitatively related to the droplet diameter *D*, the contact angle *θ*, and the refractive index contrast between the two liquids (*n*_1_ − *n*_2_), as expressed in Equation (3) [22]:

(3) $$\begin{array}{c} f=\frac{D}{2sin\theta (n_{1}-n_{2})} \end{array}$$

In addition to electrowetting, dielectrophoretic actuation, a variant of electrowetting, is compatible with a wider range of liquids and is particularly suitable for driving arrays of liquid microlenses.

In addition, another important category of interface-shape actuation is liquid-filled mechanisms. These rely on external forces such as electrostatic, electromagnetic, hydraulic, pneumatic, or thermoelectric actuation to adjust liquid volume or deform elastic membranes, which in turn changes the lens curvature and focal length. Such approaches have been extensively reviewed in previous studies [1,2], and are therefore not discussed in detail here. While liquid-filled lenses generally provide a larger tuning range, they often suffer from high driving voltages, bulky external actuators, and limited scalability, making integration into compact optical systems more challenging.

A fundamentally different approach is offered by liquid crystal (LC) lenses, which achieve variable focusing by reorienting LC molecules under external stimuli. This reorientation alters the refractive index distribution within the lens, enabling light convergence or divergence without changing the physical boundary [2,23]. Since their introduction in the 1970s, LC lenses have attracted attention for their low driving voltages, fast response, and ease of integration [24]. However, their polarization dependence, relatively high optical loss, and limited focusing range remain key drawbacks, which constrain their broader deployment.

Overall, liquid lens actuation strategies can be grouped into three major categories: electrowetting (and its dielectrophoretic variant), liquid-filled mechanisms, and liquid crystal approaches. Electrowetting is the most mature, offering fast response and simple electrical control, but is limited by contact-angle saturation and the need for conductive liquids. Dielectrophoretic actuation is compatible with a wider range of liquids and is particularly suitable for driving LMLAs. Liquid-filled mechanisms achieve wider focal ranges but at the cost of complexity and poor integrability. LC lenses, while attractive for compact, low-power applications, still face constraints due to polarization-dependent, relatively high optical loss, and a limited tuning range [1]. From an integration standpoint, electrically driven designs—especially electrowetting—remain the most practical and widely adopted solutions, while LC lenses are increasingly considered for specialized applications such as display and adaptive imaging.

Notably, core–shell double emulsion droplets composed of soft and liquid materials offer a novel route for developing liquid lenses and their arrays [25,26,27,28,29,30]. By precisely controlling the curvature of the internal liquid–liquid interface, both positive and negative focal length tuning can be achieved. In addition to retaining the rapid tuning capabilities of liquid lenses, the enclosed interfaces of these double emulsion droplets effectively package them as independent functional units, presenting substantial potential and advantages for the development of multifunctional, integrated photonic devices.

**Figure 2:**
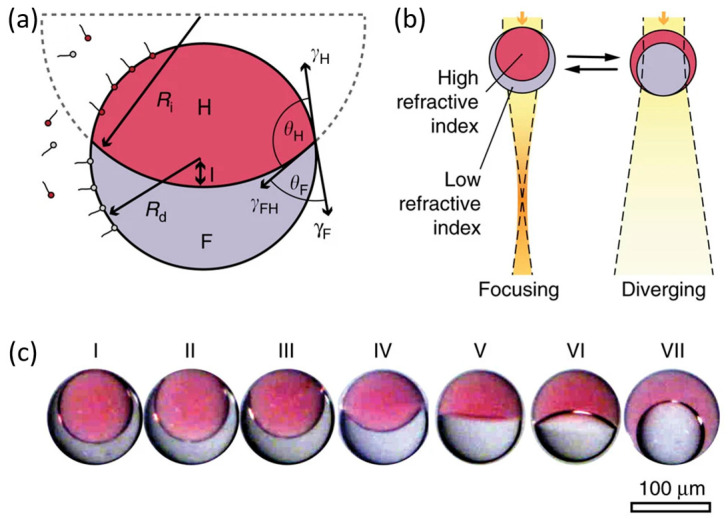
Janus double emulsion microdroplets: control mechanism, structure, and images. (**a**) Structural model of a Janus biphasic emulsion droplet [27]. (**b**) Variation in the inner interface curvature enables the droplet to converge, collimate, or diverge light [27]. (**c**) Photographs of double emulsion microdroplets [27]. All panels adapted from Ref. [27] under the terms of the Creative Commons CC BY license.

The morphology of double emulsion microdroplets is determined by the balance of interfacial tensions between the dispersed and continuous phases, with transitions among separated, dumbbell, and spherical shapes as the balance of interfacial tensions varies. The spherical configuration is known as a Janus droplet (Figure 2a), can be quantitatively described:

(4) $$\begin{array}{c} {\left(d+R_{d}-R_{i}\right)}^{2}\left(d^{2}+2d\left(R_{i}-R_{d}\right)-3{\left(R_{d}+R_{i}\right)}^{2}\right)+16d\frac{R_{d}^{3}}{1+v_{r}}=0 \end{array}$$

(5) $$\begin{array}{c} \frac{\gamma_{H}-\gamma_{F}}{\gamma_{FH}}=\frac{R_{d}^{2}+R_{i}^{2}-d^{2}}{2R_{d}R_{i}} \end{array}$$

where *R_d_* denotes the radius of the spherical droplet, *R_i_* represents the radius of curvature of the inner liquid–liquid interface, *d* is the distance from the apex of the inner interface to the droplet center, and *v_r_* is the volume ratio between the two liquid phases. *γ_H_*, *γ_F_*, and *γ_FH_* correspond to the interfacial tensions between each liquid and the surrounding continuous phase, and between the two liquids, respectively.

According to Young’s equation, the contact angle of a biphasic droplet suspended in a continuous medium is determined by the balance among these three interfacial tensions. Equation (4) defines the spherical geometry of such Janus droplets from a purely mathematical standpoint, whereas Equation (5)—derived from Young’s equation combined with the cosine rule—reveals the quantitative relationship between interfacial tensions and droplet geometry. Together, they establish the theoretical framework for understanding how interfacial forces regulate droplet morphology. Specifically, for a fixed droplet radius and phase volume ratio, the curvature of the inner interface can be tuned by adjusting the liquid–liquid interfacial tension, enabling precise modulation of the droplet’s focal length (Figure 2b).

Janus droplet lenses, based on double-emulsion structures, tune focal length via internal liquid–liquid interface modulation and are theoretically capable of offering a broader tuning range and the potential for fast response, in contrast to electrowetting lenses that rely on liquid–solid interfaces. Unlike conventional designs, Janus droplets exploit liquid–liquid optical boundaries and can be encapsulated as standalone functional units, mitigating liquid evaporation issues while providing structural stability and ease of integration. Although proof-of-concept demonstrations have been reported [26,27,28], current implementations still depend primarily on adjusting surfactant concentration in the continuous phase to alter interfacial tension and curvature, which is a slow process with limited precision and reproducibility, which restricts practical adoption. To unlock their full potential, future research should focus on developing faster and more controllable actuation strategies such as thermal, optical, or electrical stimuli, together with advances in interface engineering, fabrication, and encapsulation techniques, to enable robust, high-performance, and integrable Janus lens modules.

## 3. Materials and Fabrication Methodologies

### 3.1. Materials

In the design and optimization of liquid lenses, material selection is a core consideration. The choice of materials directly affects performance parameters, actuation mechanisms, potential applications, and fabrication strategies. From a physical perspective, the surface tension of the liquid determines the ease of interfacial deformation, which influences the precision of focal length modulation; viscosity, in turn, directly governs the response speed. Electrically, a higher liquid conductivity enables stronger charge transport, while a larger dielectric constant enhances polarization effects in non-uniform electric fields, generating stronger dielectrophoretic forces. From an optical standpoint, the refractive indices of the insulating liquid and the conductive solution must be well-matched to realize converging or diverging functionality; otherwise, aberrations may arise. Selecting liquids with high transmittance can further expand the lens’s application range. For instance, glycerol enhances tissue transparency in the near-infrared region, extending optical performance [31,32]. In terms of chemical properties, compatibility between materials and their biocompatibility are of particular importance. For example, dibutyl adipate (Tokyo Kasei Kogyo, Japan) can be brought into direct contact with electrode materials without requiring an insulating coating [11]. For liquid-crystal-based lenses, key parameters include the elastic constants, anchoring energy, dielectric anisotropy, refractive index anisotropy, and temperature dependence [33].

In practice, the materials used in liquid lenses can be broadly categorized into four groups, which collectively determine device performance and applicability. First, electro-or dielectric-responsive materials enable refractive index modulation or interface reconfiguration under applied electric fields. Representative examples include conductive electrolytes (NaCl (EMD Chemicals, Darmstadt, Germany), KCl solutions), ionic liquids (BMIMBF_4_, EMIMBF_4_), liquid crystals (E7 (Daily Polymer Kaohsiung, Kaohsiung, Taiwan, China), 5CB), and polar or high-dielectric liquids such as propylene carbonate (PC) and low-molecular-weight silicone oils. These materials achieve optical modulation by altering droplet interfaces or molecular orientation, serving as the core components for electrowetting and electrically controlled liquid lenses. Their properties, such as conductivity, dielectric constant, and freezing point, directly influence response speed, actuation efficiency, and operational temperature range.

As shown in Table 1 and Table 2, water-based conductive liquids have low viscosity, which enables a fast response, coupled with high conductivity, making them the most common working fluids in electrowetting lenses. However, they are prone to electrolysis and bubble formation, which may shorten device lifetime. By contrast, ionic liquids and electroresponsive fluids are resistant to electrolysis, enabling stable long-term operation. Electroresponsive fluids such as tetradecanedioic acid can be actuated directly under DC fields and interfaced directly with electrode materials, greatly simplifying device fabrication. In dielectrophoretic liquid lenses, ionic liquids with large differences in dielectric constant and refractive index are often paired to generate dielectrophoretic forces under polarized fields. Moreover, their freezing points can be tailored through molecular design: EMIM BF_4_ with a short ethyl side chain has a higher freezing point than analogs with longer alkyl side chains (BMIMBF_4_), because increased chain length reduces molecular symmetry and weakens intermolecular interactions. With wide operational temperature ranges and tunable freezing points [10], ionic liquids are advantageous for extreme environments.

Second, optical functional media are typically insulating liquids that provide refractive index contrast, tunable birefringence, or optical anisotropy. Representative examples include immiscible liquid pairs and base liquids such as glycerol, silicone oil (PMX-200 (The Dow Chemical Company, Midland, MI, USA)), alkanes (dodecane, ethane, hexane), fluorocarbon oils (perfluorocarbons, perfluorohexane, commercial mixtures such as A58-N1-P37), propylene carbonate (PC), and liquid crystals (E7, 5CB). Their primary role is to establish refractive index differences for optical functionality rather than directly responding to electric fields. Liquids like alkanes and glycerol can serve both as insulating phases in electrically actuated lenses and as fillers in fluidic lenses.

Third, encapsulation and structural materials provide mechanical support, chemical resistance, and optical transparency. Examples include elastomers (dielectric elastomers, liquid crystal elastomers), hydrogels, high-molecular-weight polymers (PDMS (The Dow Chemical Company, Midland, MI, USA), PMMA, COC, PVC, PU), and UV-curable polymers (NOA65, NOA78, NOA81) (Norland Products, Inc., Cranbury, NJ, USA). These materials maintain the structural integrity of liquid lenses while enabling controlled deformation when required. The elastic modulus is a key parameter for evaluating mechanical stability: a lower modulus allows greater deformation, which in turn affects shape stability and focal precision. However, elasticity is not constant and can vary with humidity, temperature, and doping ratio [45,46]. As summarized in Table 3, dielectric elastomers exhibit diverse trade-offs. For example, Very High Bond (VHB) acrylic elastomers have a narrow operational temperature range and are prone to performance fluctuations at extreme temperatures; silicone rubber offers excellent thermal stability, chemical stability, and biocompatibility for long-term operation under appropriate conditions, though its low dielectric constant necessitates high driving voltages; polyurethane dielectric elastomers, by contrast, possess high dielectric constants and thus generate strong actuation forces, but are sensitive to environmental humidity, with Young’s modulus variations of up to ~0.6 MPa between dry and humid states, leading to performance instability.

Liquid crystal elastomers (LCEs) retain intrinsic liquid crystal properties—responding to optical, electrical, and thermal stimuli—while providing reversible elastic deformation. They enable remote light actuation and high energy storage–release efficiency [51]. Hydrogels, widely used in bio-inspired actuators, can achieve strain, strength, and response speeds comparable to biological muscles. Their elasticity can be tuned by adjusting plasticizer content, though this raises biocompatibility concerns. For these elastomeric materials, incorporating high-k nanoparticles can increase dielectric constants, which reduces driving voltage [46]. Similar strategies also improve mechanical and thermal stability, as well as responsiveness to optical, thermal, magnetic, and electrical stimuli, enhancing environmental adaptability and broadening applications [13,14,45,52]. Combined with additive manufacturing, such materials can be leveraged for 4D printing, microlens arrays, and complex three-dimensional architectures [53,54].

Four, auxiliary functional materials improve stability, wettability, or response dynamics. Examples include surfactants (SDS, CTAB, Span 80), nanoparticles (SiO_2_, TiO_2_, BaTiO_3_), dyes, dielectric or insulating layers (parylene HT, fluorinated polyimides), and surface treatment layers (PVA, Teflon, CYTOP). They regulate droplet interfaces, suppress coalescence or scattering, and enhance overall optical and electrical performance. Such materials complement the core functional and structural materials, further broadening environmental adaptability and tuning response dynamics.

Considering overall material performance and functionality, electrically actuated liquid lenses currently represent the most compromise and have been extensively studied in consumer electronics, microscopy, and display technologies. Dielectrophoretic liquid lenses, leveraging non-uniform electric fields, lay the foundation for realizing aspherical profiles and thus hold advantages in aberration correction. Hybrid actuation mechanisms that combine different driving strategies are especially suited for high-precision imaging applications. By contrast, fluidic lenses are generally bulky and less compatible with miniaturized devices; moreover, some materials require high operating voltages, limiting their practicality in mainstream mobile electronics.

### 3.2. Fabrication Method

**Figure 3:**
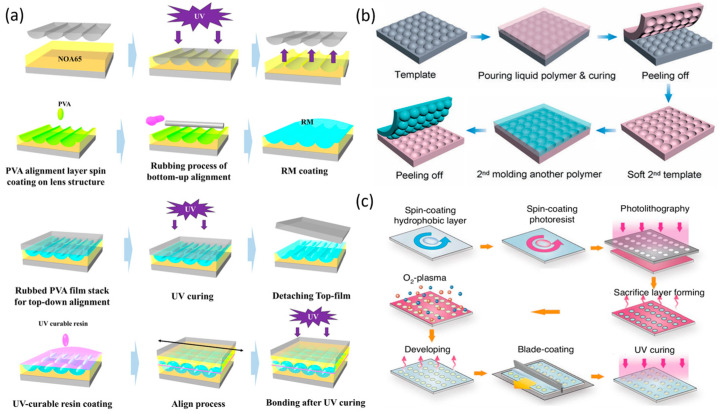
Illustration of semiconductor-derived processes. (**a**) Fabrication of LC-MLAs via nanoimprinting: PVA spin-coated and rubbed to control LC orientation [62]. (**b**) Two-step soft lithography replication: first replication produces inverse structure, second step yields positive copy [63]. (**c**) LMLAs via selective wettability: oxygen plasma modifies the hydrophobic layer, exposing hydrophilic regions [64]. All panels adapted from Refs. [62,63,64] under the terms of the Creative Commons CC BY license.

At present, semiconductor-derived processes represent the mainstream fabrication technology for LMLAs, meeting the demands for optical precision, high uniformity, and scalability in mass production. Structurally, LMLAs consist of several key functional layers. Electrodes and dielectric layers are typically deposited via physical or chemical vapor deposition (PVD or CVD), while large-area hydrophilic and hydrophobic coatings are applied through spin coating or similar deposition techniques. Patterning is achieved using photolithography and etching. The fabrication of both liquid microlens arrays (LMLAs) [55,56] and liquid crystal microlens arrays (LC-MLAs) [57,58] follows essentially the same sequence as single lenses [59], relying on deposition, photolithography, etching, and coating. For LC-MLAs, alignment layers are spin-coated and subjected to rubbing treatment to ensure uniform molecular orientation (Figure 3a) [60,61].

In addition, array structures on target material surfaces can be realized using alternative techniques such as soft lithography, replica molding, nanoimprinting, and reactive ion etching (RIE). Soft lithography, replica molding, and nanoimprinting all employ mold-replication strategies. In soft lithography, an elastic mold transfers micro- and nano-scale features to the target surface via contact or imprinting (Figure 3b) [65]. Replica molding fills a mold with the target material and cures it to directly form the desired arrays [66]. nanoimprinting presses a mold into a thermoplastic polymer, followed by thermal or UV curing and demolding to replicate structures (Figure 3a) [62]. RIE, by selectively modifying the substrate surface, leverages differences in surface wettability to induce material self-assembly in hydrophilic regions (Figure 3c) [64,67,68]. These approaches collectively provide versatile and cost-effective routes for producing high-quality LMLAs on a large scale.

**Figure 4:**
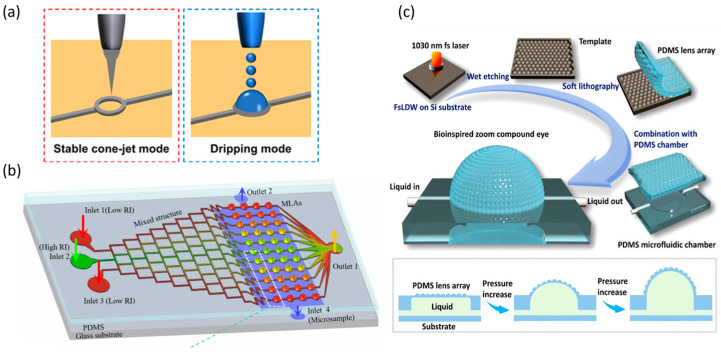
Additive manufacturing approaches. (**a**) Electrodes and liquid microlenses are fabricated by electrohydrodynamic jet (E-jet) printing: print microelectrodes in a stable cone-jet mode and print liquid microlenses in dripping mode [71]. (**b**) A microstructure model is fabricated using two-photon 3D printing; PDMS is poured into the molds and removed to yield microfluidic chips embedded with a microcavity array [72]. (**c**) Array structure molds are fabricated using a femtosecond laser; PDMS is poured, removed, and shape-set cured to obtain curved compound-eye arrays [73]. All panels adapted with permission from [71,72,73]. Copyright American Chemical Society.

Additive manufacturing techniques, including three-dimensional (3D) printing, inkjet printing, and direct laser writing, enable the fabrication of complex 3D asymmetric structures through point-by-point or layer-by-layer processes [69]. By eliminating the need for molds, these methods accommodate intricate lens geometries and facilitate flexible, customized production. Three-dimensional printing builds structures from digital models via successive layer-by-layer material deposition [70]. Inkjet printing deposits liquid ink onto the substrate as droplets through a nozzle (Figure 4a). Direct laser writing employs a focused laser beam to create microstructures on or within a material through point-by-point or line-by-line exposure. In practical applications, additive manufacturing methods, such as 3D printing and direct laser writing, are often combined with semiconductor-derived processes (Figure 4b,c).

Dynamic shaping technologies leverage external fields (light, electric, or thermal) to modulate material phase and flow properties in situ, offering distinct advantages in programmable manufacturing. The core advantage lies in exploiting the self-leveling behavior of liquid interfaces to achieve high-precision fabrication; however, scaling these processes demands addressing challenges related to interface stability. Representative techniques include droplet microfluidics, liquid–liquid phase separation, and laser injection.

**Figure 5:**
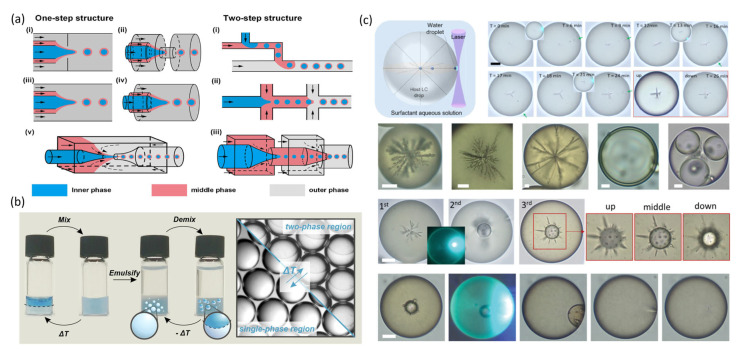
Dynamic shaping techniques. (**a**) Generation of double emulsion microdroplets using microfluidic approaches [75]. Adapted with permission from [75]. Copyright 2024 American Chemical Society. (**b**) Formation of double emulsion microdroplets through temperature-driven liquid–liquid phase separation [79]. Adapted from Ref. [79], with permission from Elsevier. (**c**) Laser injection techniques, encompassing both the injection and coalescence processes [6]. Adapted from Ref. [6] under the terms of the Creative Commons CC BY license. Scale bars: the black one is 20 μm and the white one is 10 μm, respectively.

Droplet microfluidic techniques enable controlled droplet generation through carefully designed microchannel architectures [74,75,76]. As illustrated in Figure 5a, the fundamental mechanism involves the interfacial tension balance between immiscible liquids under shear or compression, leading to the spontaneous formation of biphasic or multiphase spherical droplets. Droplet size can be tuned by channel dimensions and flow rates, while parallelization of channels increases production throughput, and cascaded channels enable multi-step emulsification. Incorporating liquid–liquid phase separation within microchannels further facilitates the assembly of complex droplet structures [77,78]. Temperature-driven phase separation relies on controlling the miscibility of two oil phases: heating yields a homogeneous phase, whereas cooling induces droplet formation (Figure 5b). Adding surfactants allows dynamic transformation between core–shell and Janus droplet morphologies, supporting diverse optical focusing requirements [79].

Laser injection techniques allow droplet fabrication without physical nozzle contact and comprise three processes: laser injection, laser-induced coalescence, and laser-induced reconstruction [80]. A focused laser beam serves as a localized heat source to induce nanodroplet injection on the surface of LC droplets via the photothermal effect. Precise control of the laser beam and irradiation position governs the self-assembly and merging dynamics of the injected water nuclei, enabling high-precision fabrication of customized 3D structures of liquid crystal droplets (Figure 5c). Although still in early development, this bottom-up approach offers substantial potential for tailoring the internal architecture of droplets.

## 4. Core Functions

### 4.1. Focal Length Tuning

Focal length tuning is a fundamental feature of liquid lenses, achieved by modulating either the interface curvature or the refractive index of LC materials to control light convergence and divergence, thus enabling continuous adjustment of the focal length. Rapid focusing is crucial for applications such as machine vision and industrial inspection, and researchers have continually sought to enhance both the tuning range and response speed of liquid lenses to accurately capture information from objects at varying spatial locations, supporting practical applications.

**Figure 6:**
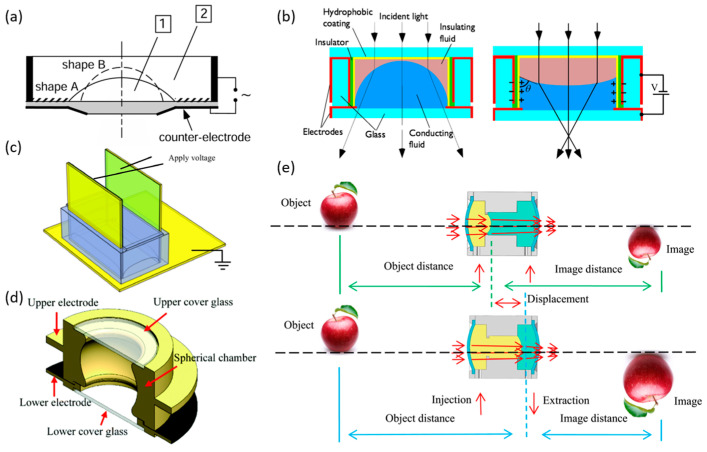
Liquid lens configurations. (**a**) Planar hemispherical liquid lens supports unidirectional focal tuning [81]. Adapted from Ref. [81] with permission from Springer Nature. (**b**) Cylindrical liquid lens, allowing bidirectional tuning [82]. Adapted from Ref. [82] with permission from AIP Publishing. (**c**) Rectangular-cavity liquid lens [83]. Adapted with permission from Ref. [83] © Optical Society of America. (**d**) Spherical cavity liquid lens [84]. Adapted from Ref. [84], with permission from the Royal Society of Chemistry. (**e**) Liquid lens with a movable liquid–liquid interface, enabling zoom for sharp images at varying magnifications [85]. Adapted from Ref. [85], with permission from Elsevier.

During the early stages of liquid lens development, a major breakthrough was made in extending the focal length tuning capability from unidirectional to bidirectional operation. Initially, liquid lenses were limited to unidirectional focal length tuning (Figure 6a). Berge et al. utilized the electrowetting effect to control a planar hemispherical liquid lens, achieving continuous focal length adjustment from 16 mm to 10 mm with a response time of approximately 30 ms [81]. Because the lens surface remained consistently convex, it could only converge light, resulting in unidirectional focal length tuning. To enable bidirectional focal length tuning, Kuiper et al. introduced the classic cylindrical liquid lens (Figure 6b), whose interface can switch between concave and convex profiles. The lens achieves minimum positive and negative focal lengths of +20 mm and −10 mm, respectively, with a maximum extending to infinity [82]. The cylindrical configuration has since become the most commonly adopted design for liquid lenses.

To further broaden the tuning range and enhance response speed, research has primarily focused on three key strategies: expanding the contact angle modulation range, improving the stability of the liquid materials, and lowering the driving voltage. Cavity geometry innovations have significantly increased tuning ranges and zooming degrees of freedom while addressing sidewall roughness and contact–angle limitations. Wang et al. substituted the conventional cylindrical cavity with a rectangular one (Figure 6c), broadening the focal length tuning range [83]. Xu et al. adopted a novel strategy, introducing a spherical-cavity electrowetting liquid lens (Figure 6d) that expanded the contact angle range from 34 to 144° in the traditional cylindrical design to 14–166°, achieving a focal length range of −72.3 mm to +70.3 mm [84]. They also proposed a sandwich configuration based on this spherical cavity—conductive liquid–insulating liquid–conductive liquid-featuring two liquid–liquid interfaces, which enhances the lens’s zooming degrees of freedom [86]. To create liquid lens cavities of diverse geometries, Chen and colleagues developed an EWOD-based winding technique, in which insulated flexible wires are tightly coiled to form the lens cavity, with the electrodes and dielectric layer patterned in a single-step process [87]. Building on this approach, in 2025, they proposed a dual-cavity configuration that separates the driving and optical cavities. This design not only addresses the rough sidewall issue associated with the original method but also breaks the contact angle constraint on the focal length tuning range [88].

In addition to modifying cavity designs, various strategies have recently been developed to extend the focal length tuning range. Li et al. introduced an electrowetting liquid-piston-based optofluidic lens to broaden the tuning range [89]. Kopp and Huang et al. incorporated two liquid–liquid interfaces into a sealed cylindrical cavity and a planar hemispherical liquid lens, respectively. The independent control of these interfaces enabled substantial focal length variation [90]. Additionally, employing a PDMS membrane to modulate the optical interface of a liquid lens circumvents the contact angle saturation and hysteresis limitations of electrowetting, offering a novel approach to further extend the focal length tuning range [91].

By controlling the position and morphology of the liquid–liquid interface, liquid lenses can achieve independent zooming while maintaining sharp focus, which simplifies optical system design. Conventional single liquid lenses tune their focal length solely through interface curvature, but the continuous back-focal-length variation limits their standalone zoom capability. In contrast, moving the liquid–liquid interface adjusts object and image distances to reach the desired magnification, followed by interface reshaping to refocus the image. Zhang et al. demonstrated precise control in a liquid interface to achieve single-lens optical zoom (Figure 6e), significantly streamlining zoom system architectures [85].

**Figure 7:**
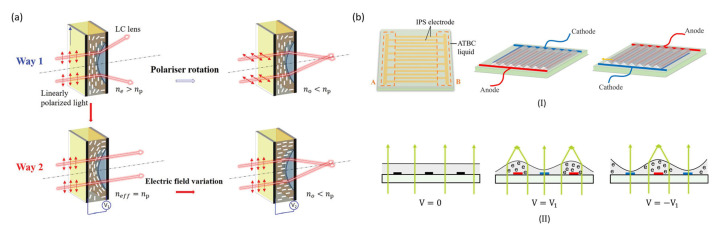
Focal length tuning in LMLAs. (**a**) The LC-MLA mentioned above can modulate light convergence and divergence via two methods: placing polarizers or applying a voltage [92]. Adapted from Ref. [92], with permission from Taylor & Francis. (**b**) Electrically responsive fluid liquid microlens array (**I**) Application of a reverse voltage enables movement of the Electrically responsive fluid liquid microlens array (**II**) Shifting the liquid layer allows the lens to converge and diverge light at the same location [93]. Adapted from Ref. [93], with permission from Elsevier.

LMLAs offer low distortion, minimal aberrations, wide viewing angles, and effectively infinite depth of field, attracting considerable research interest. LC-MLAs operate at low driving voltages. In 2021, Xue et al. developed an LC-MLA (Figure 7a) capable of continuous focal length adjustment from −4 mm to +6 mm under a voltage range of 0.7–10 Vrms [92]. However, the use of polarizers results in ~50% light loss, and the low birefringence of liquid crystals limits refractive index modulation, constraining focal tuning. Electrowetting-based LMAs provide compact designs, such as Kim et al.’s 3 mm-thick LMLA combined with solid microlenses, achieving a fill factor increase from 70% to 100% in tunable integral imaging systems [55]. Xu et al. demonstrated an electrically responsive fluid-based LMLA with continuous tuning from infinity down to 180 μm (Figure 7b). This design, relying solely on patterned electrodes and responsive fluid, is lighter and thinner than LC- or electrowetting-based LMLAs, and electrically responsive fluid resists ionization and hydrolysis, addressing electrowetting limitations [93]. In 2024, the same group further developed an LMA in which glycerol curvature is modulated via dielectrophoretic forces for precise focal control [94].

### 4.2. Field of View Expansion

In optical systems, the FOV defines the spatial region that can be observed, typically quantified by the maximum angular range captured in a given direction. The FOV is inversely related to focal length, and the continuous optical zoom capability of liquid lenses inherently enables FOV adjustment. Nonetheless, to satisfy the demands of large-FOV applications such as endoscopy, performance must be further enhanced and optimized. Two primary approaches have been proposed to expand the FOV of liquid lenses: one involves controlling the interface deflection to redirect the optical axis, which in turn extends the original FOV; the other employs MLAs to achieve FOV expansion.

**Figure 8:**
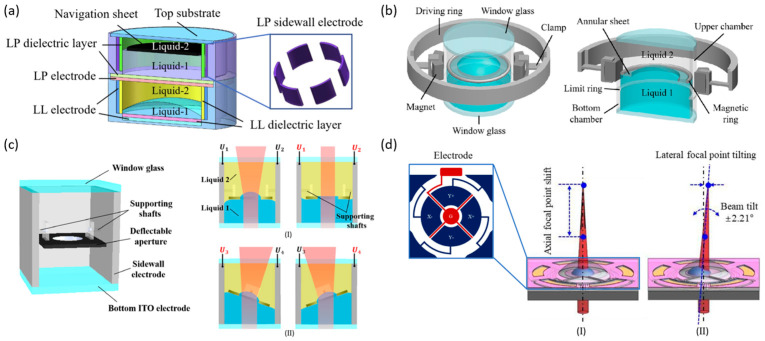
Structures and mechanisms for FOV steering in liquid lenses. (**a**) Multifunctional optofluidic lens configuration [96]. (**b**) Liquid lens with a single liquid–liquid interface enabling simultaneous focal length tuning and FOV steering [97]. (**c**) Four-electrode-controlled liquid lens demonstrating divergent, collimated, and left/right FOV deflection states [98]. (**d**) Planar hemispherical liquid lens driven by five electrodes, showing axial and lateral focal shifts [99]. All panels adapted with permission from Refs. [96,97,98,99] © Optical Society of America.

To enable FOV deflection, Takei et al. combined a tunable water prism and an oil lens into a single optical system using electrowetting technology. Focal length adjustment was achieved by adjusting the curvature of oil droplets within the water phase, while FOV deflection of 6.3° was accomplished by modifying the contact angle between the plate and the water droplet as it moved [95]. Liu et al. presented a multifunctional optofluidic lens (Figure 8a), comprising a stacked liquid lens cavity and liquid prism cavity. The dual-cavity separated design ensures independent operation of optical focusing and beam steering. In the bottom cavity, the liquid–liquid interface curvature is controlled via electrowetting, whereas in the upper cavity, six electrodes drive a navigation plate, allowing the beam deflection angle to be continuously tuned from 0° to 22.8° [96]. To simplify system architecture, Tian et al. and Yin et al. demonstrated liquid lenses (Figure 8b,c) in which a single liquid–liquid interface enables simultaneous focal length adjustment and FOV deflection [97,98]. In 2025, Seo et al. introduced a five-electrode design to steer a planar hemispherical liquid lens (Figure 8d) and successfully integrated it into a photoacoustic endoscope probe, achieving field-of-view expansion without the need for mechanical scanning [99].

Furthermore, integrating liquid lenses with fisheye or other solid lenses can further enhance the FOV in practical applications. Zohrabi et al. introduced an innovative approach combining multiple tunable liquid lenses with fisheye lenses to achieve wide-angle, non-mechanical beam steering, accomplishing ±75° two-dimensional beam deflection [100]. Choi et al. proposed an ultra-wide-field optical system for optical detection and ranging by integrating liquid lenses with commercial fisheye lenses, and employed relay optics to efficiently couple the intermediate image planes of the two lens systems, enabling sequential scanning of high-power laser beams and achieving an ultra-wide FOV of approximately 170° [101].

**Figure 9:**
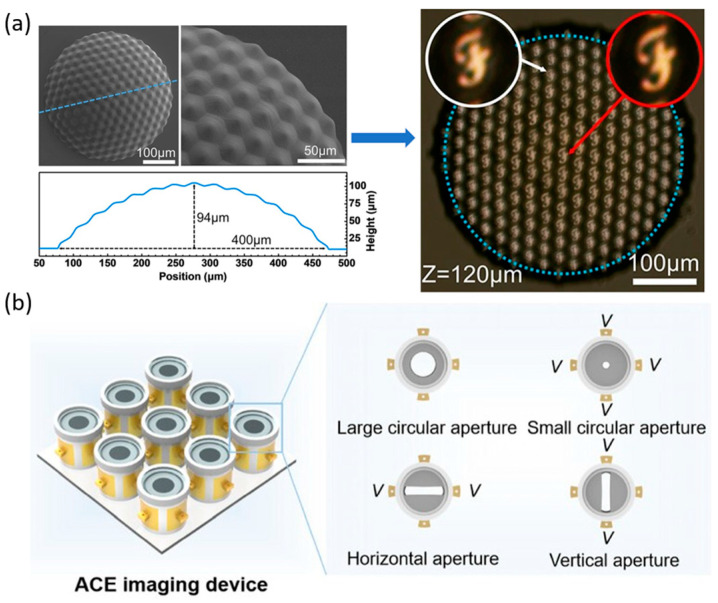
Structures of LMLAs’ compound-eye systems. (**a**) SEM image of a fixed-FOV compound-eye camera and its corresponding imaging results [104]. (**b**) Adaptive compound-eye imaging device based on a LMLA [105]. All panels adapted from Refs. [104,105] under the terms of the Creative Commons CC BY license.

Compared with a single lens, microlens arrays (MLAs) as multi-aperture optical systems provide significant FOV expansion [102]. Curved MLAs are inspired by insect compound eyes, where each ommatidium captures light from a different angle, and spatial arrangement enables wide-area imaging through FOV stitching. A miniature optoelectronic compound-eye camera (Figure 9a) demonstrated this principle with a fixed overall viewing angle of 90°, while liquid-filled and shape-memory-polymer-based compound-eye systems further enabled tunable curvature, achieving adjustable FOVs up to 160° [67,103,104]. However, in these designs, the focal lengths of individual ommatidia remain fixed, leading to blurred peripheral views and restricting clear imaging to a single depth. To overcome this limitation, an adaptive compound-eye imaging system based on electrowetting liquid lenses was recently developed (Figure 9b), together with a sparse compound-eye camera combining fast optical zoom with computational decoding, achieving high-resolution, adaptive wide-FOV imaging [105,106].

### 4.3. Depth of Field Extension

DOF defines the spatial range within which objects appear sharply focused, directly influencing both image quality and the integrity of captured information. Research on liquid lens DOF has primarily centered on microscopic imaging, holographic imaging, and 3D display applications, including non-contact dermoscopy, optical coherence tomography of blood vessels, and three-dimensional measurements [107,108].

Strategies for extending DOF can be classified into direct hardware-based approaches and indirect algorithm-based approaches. Indirect approaches rely on computational imaging algorithms to optimize image quality, whereas direct approaches modify the optical components themselves. A widely used optical strategy is to increase imaging resolution. As expressed in Equations (6)–(8) [109], DOF, minimum resolvable distance (*σ*), and numerical aperture (*NA*) are interdependent: DOF decreases with increasing *NA* and *σ*. Extending DOF thus requires reducing the *NA*, which requires reducing *Φ* and simultaneously increasing *σ*, ultimately lowering resolution. This reflects the intrinsic trade-off in optics—achieving a larger DOF inevitably compromises resolution and detail fidelity. While this limitation cannot be completely overcome, enhancing resolution through alternative techniques can improve the perceived depth of field, which effectively meets application requirements.

(6) $$\begin{array}{c} DOF=\frac{\lambda }{{NA}^{2}}+\frac{\delta }{M\cdot NA} \end{array}$$

(7) $$\begin{array}{c} NA=\frac{\emptyset }{2f} \end{array}$$

(8) $$\begin{array}{c} \sigma =\frac{0.61\lambda }{NA} \end{array}$$

Here, *NA* the numerical aperture; *λ* is the operating wavelength; *δ* the pixel size of the camera; *M* the magnification of the microscope; ∅ the aperture stop diameter; *f* represents the object objective focal length; *σ* denotes the minimum resolvable distance, where a smaller *σ* indicates higher resolution.

The composite strategy of combining dynamic focusing via liquid lenses with data fusion has become a widely adopted method for indirect DOF extension. In this approach, an electrically tunable liquid lens sequentially adjusts its focal depth to capture multi-plane images, which are then fused algorithmically to generate an extended-DOF image and enable rapid depth estimation [110]. Building on this, Ping et al. integrated lightweight deep learning models [111], achieving a depth range of up to 23 mm and completing DOF-extended imaging of 144 MB images within 1 s. During image acquisition, the liquid lens can either (i) dynamically vary its focal position within a single exposure, producing an integrated image that encodes blurred information from the entire focal range, or (ii) capture multiple sharp images at different focal depths, which are subsequently fused into a single sharp extended-DOF image. The trained model learns to map defocused inputs to clear, extended-DOF outputs, enabling real-time reconstruction of sharp images from blurred data. This approach effectively balances imaging quality and processing efficiency, meeting the real-time demands of industrial inspection.

**Figure 10:**
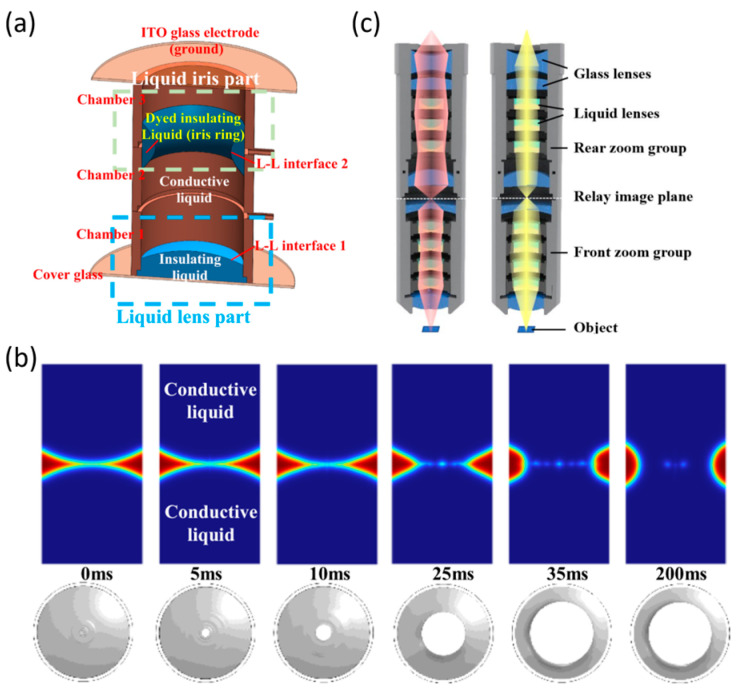
Applications of liquid lenses in DOF extension. (**a**) Integrated design combining zoom and aperture control [112]. (**b**) Illustration of aperture variation [112]. Adapted from Ref. [112], with permission from Elsevier. (**c**) Multiple liquid lenses enabling large-DOF microscopic imaging without mechanical adjustment [113]. Adapted from Ref. [113] under the terms of the Creative Commons CC BY license.

The aperture size and DOF are inversely related, allowing DOF to be directly controlled (Equations (6) and (7)). Liquid apertures have progressed from variable liquid diaphragms, analogous to traditional mechanical apertures, to multifunctional liquid lenses integrating both focal length and aperture adjustment [59,112]. To overcome limitations of conventional designs, where electrowetting liquid lenses and liquid irises were separated, leading to structural complexity and irregular aperture shapes, Xu et al. proposed an integrated configuration [112]. In their design, the lens and iris share a conductive liquid: the red region represents the insulating liquid, and the blue region the conductive liquid (Figure 10a). By tuning the distance between two liquid–liquid interfaces formed by three immiscible liquids, the merged interfaces generate a liquid ring adhering to the chamber wall (Figure 10b). This integration eliminates the complexity of traditional separate designs and enhances DOF tunability.

To reconcile the trade-off between large DOF and high resolution in microscopy, hybrid strategies that combine direct and indirect approaches have been explored. Liu et al. employed seven liquid lenses working collaboratively to achieve axial scanning without mechanical movement, while an improved Laplacian pyramid fusion algorithm mitigated image blurring. Their system consists of two magnification units (front and rear groups), each containing one primary tunable lens and auxiliary lenses for aberration correction (Figure 10c). This design achieves continuous magnification from 10× to 60× with resolution exceeding 512 lp/mm [113]. Zhang et al. enhanced perceived DOF by combining annular illumination with Richardson–Lucy deconvolution, effectively suppressing background noise and improving image clarity [114]. Cai et al. proposed a structured illumination experimental scheme and computational framework incorporating two electrically tunable lenses, which extends the DOF of structured light systems. Extended the effective DOF to 8 mm and achieved more than a fivefold performance improvement compared with the original system [115].

The principle of DOF extension in LC-MLAs lies in superimposing the depth range of multiple sub-lenses [116,117]. Unlike MLAs, with fixed focal lengths, LMLAs and LC-MLAs enable dynamic focal switching or distribution.

**Figure 11:**
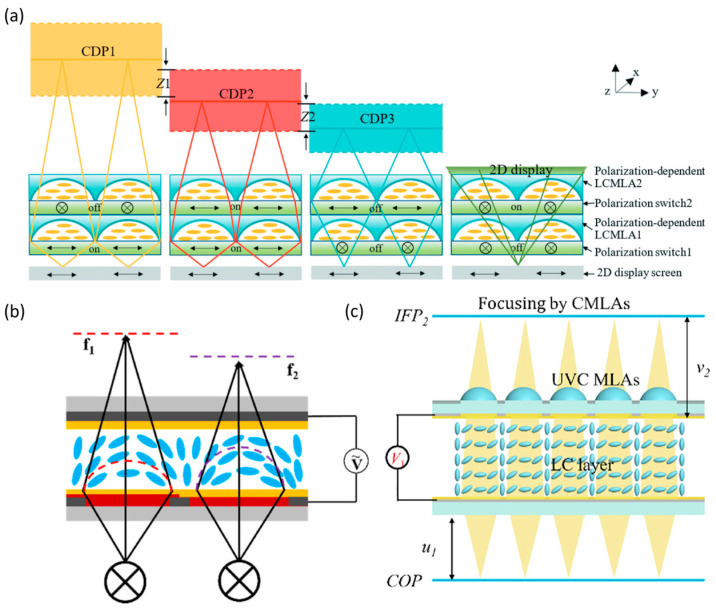
Implementation of multiple depth planes in LC-MLAs. (**a**) Polarization switching independently controls the x/y polarization states of incident light in the top and bottom LC-MLAs layers, enabling focusing or transmission; from left to right, the sub-depths are 25 mm, 17 mm, 9 mm, and 0 mm [118]. Adapted from Ref. [118], with permission from Taylor & Francis. (**b**) Sub-lenses with different resistive layer thicknesses exhibit distinct voltage responses: a thicker resistive layer induces a weaker response, larger curvature, and longer focal length (left: 40 nm; right: 30 nm) [119]. (**c**) Hybrid configuration combining LC-MLAs with UV-cured MLAs [60]. Adapted from Refs. [60,119], with permission from Elsevier.

For LC-MLAs, DOF can be extended by stacking multiple layers, typically two. Focal length differences are realized either by modulating the polarization states of the incident light, where linearly combining focusing and transmission modes yields four distinct central depth planes (CDPs) (Figure 11a) [118], or by tailoring the stress response of the lenses. Zhang et al. introduced resistive layers of varying thickness, producing distinct electric field distributions under the same voltage, thus realizing two focal lengths (Figure 11b), and extending DOF to two to three times that of conventional LC-MLAs [119]. Wang et al. further enhanced focusing stability by combining LC-MLAs with fixed-focal-length UV-cured MLA (Figure 11c), achieving a maximum DOF of 43.38 mm, significantly surpassing the 23.85 mm of a single LC-MLA [60].

### 4.4. Aberration Correction

**Figure 12:**
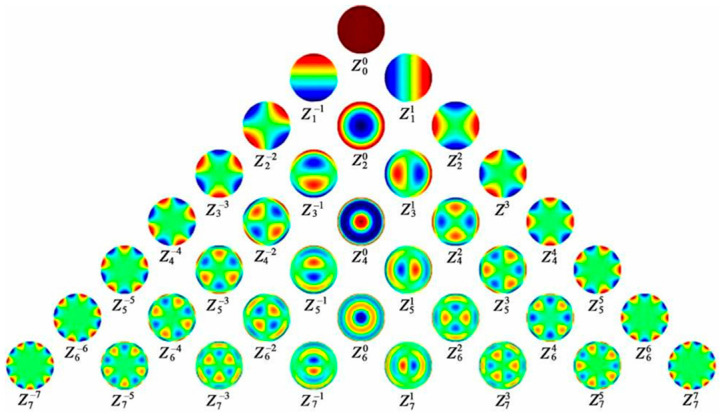
Wavefront shapes of the first eight Zernike polynomial orders [122]. Adapted from Ref. [122] under the terms of the Creative Commons CC BY 4.0 license.

Aberrations fundamentally constrain the sharpness, color fidelity, and spatial accuracy of imaging, serving as a key metric for evaluating the performance of liquid lenses. They describe the deviation of an optical system’s actual imaging from its ideal counterpart. Complex wavefront aberrations are typically decomposed into a linear combination of Zernike polynomials [120], as shown in Equations (9) and (10) [121]. The corresponding coefficients $c_{n}^{m}$ quantify the magnitude of each type of aberration, $R_{n}^{m}\left(\rho \right)$ as the radial polynomials. Similarly, aberration patterns can also be reconstructed from a specific set of Zernike polynomials [121]. Based on symmetry, aberrations can be classified into rotationally symmetric terms (*m* = 0), such as defocus and spherical aberration, and non-rotationally symmetric terms (*m* ≠ 0), like astigmatism and coma (Figure 12).

(9) $$\begin{array}{c} W=\sum_{n≧0}\sum_{|m|≦n}c_{n}^{m}Z_{n}^{m}\left(\rho ,\theta \right) \end{array}$$

(10) $$\begin{array}{c} Z_{n}^{m}\left(\rho ,\theta \right)=\left\{\begin{array}{c} R_{n}^{m}\left(\rho \right)cos\left(m\theta \right),m≧0\\ R_{n}^{\left|m\right|}\left(\rho \right)sin\left(|m|\theta \right),m<0 \end{array}\right. \end{array}$$

The predominant aberrations in liquid lenses include chromatic aberration, spherical aberration, among others. Their correction is typically guided by third-order aberration theory and thin-lens theory [123]. The process involves constructing a system of multi-constraint equations by setting the total aberration coefficients to zero, applying customized constraints to derive the optimal solution parameters, and subsequently adjusting lens parameters to achieve aberration correction. More complex mixed aberrations are typically addressed using a system that integrates real-time measurement, feedback, and compensation [124]. Additionally, optical design tools such as ZEMAX and MATLAB facilitate simulation and performance evaluation. Aberration correction is commonly assessed using the Root Mean Square (RMS) and the Strehl Ratio (SR) metrics [125]. A smaller RMS value indicates less deviation from the ideal form, reflecting better aberration correction. The SR (0 < SR < 1) measures image quality, with higher values indicating a more perfect image; generally, an SR above 70% is considered acceptable. The following sections provide a detailed discussion of the main aberration types in liquid lenses and their corresponding correction strategies.

#### 4.4.1. Chromatic Aberration

**Figure 13:**
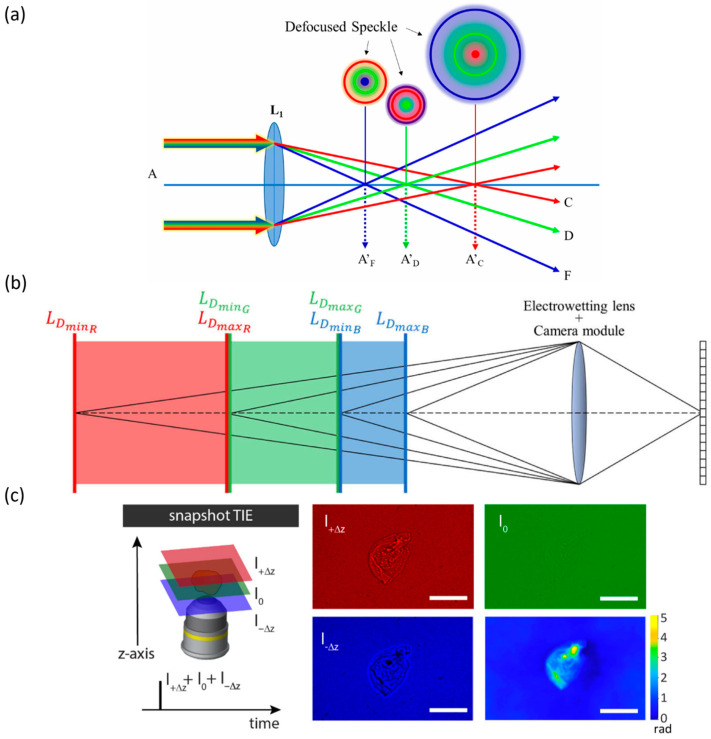
Definition, correction strategies, and practical applications of chromatic aberration. (**a**) Optical paths of chromatic aberrations [133]. Adapted from Ref. [133] under the terms of the Creative Commons CC BY license. (**b**) Depth information across three color channels obtained using a liquid lens [134]. Adapted with permission from Ref. [134] © Optical Society of America. (**c**) Phase imaging achieved using a TAG lens [135]. Adapted from Ref. [135] under the terms of the Creative Commons CC BY license. Scale bars: 100 μm.

In an optical system, light of different wavelengths cannot be focused at a single point due to variations in refractive index, a phenomenon known as chromatic aberration. This effect is intrinsic to all optical materials, including liquids and glass. Figure 13a illustrates that blue light, having a higher refractive index than red light, focuses closer to the lens. At the blue focal plane, red and green light remain defocused, producing blurred spots in the image. In a single lens, a convex lens focuses blue light closer to the lens and red light farther away, while a concave lens behaves oppositely; combining the two can achieve complementary dispersion. To satisfy the overall converging/diverging power and ensure proper imaging, the lens curvatures must be adjusted so that the total optical power is matched. The classical achromatic scheme is the doublet lens, in which a low-dispersion convex lens is paired with a high-dispersion concave lens to bring different wavelengths of light to the same focal point—this represents the fundamental principle of chromatic aberration correction. The theory of chromatic aberration correction in liquid lenses is consistent with that of conventional lenses, but the tunable interfaces and selectable materials of liquid lenses provide greater flexibility. In this context, the achromatic condition must be satisfied as follows [126]:

(11) $$\begin{array}{c} \varphi =c\left(n-1\right) \end{array}$$

(12) $$\begin{array}{c} v=\frac{n_{d}-1}{∆n} \end{array}$$

(13) $$\begin{array}{c} \sum_{k=1}^{l}\frac{\varphi_{k}}{v_{k}}=0 \end{array}$$

Here, *φ* denotes the optical power of a single lens, *n* is the refractive index, and *c* is the lens curvature (*c = c*_1_ − *c*_2_, where *c*_1_ and *c*_2_ are the curvatures of the spherical surfaces). The dispersion of the optical material is characterized by the Abbe number *v*, where *n_d_* is the refractive index with respect to the yellow Fraunhofer d-line, and Δ*n = n_F_* − *n_C_* is the mean dispersion. *φ_K_* corresponds to the optical power of the k-th lens, and *v_K_* indicates its Abbe number.

In terms of cost and optical performance, liquid materials are better suited than glass for apochromatic designs. Traditional achromatic doublets consist of a low-dispersion convex crown glass element and a high-dispersion concave flint glass element, correcting chromatic aberration at two selected wavelengths, typically red and blue. Apochromatic lenses further compensate for focal shifts at intermediate wavelengths, usually green light, requiring carefully selected material combinations and precise control of their dispersion properties. Robert D. Sigler analyzed 300 optical liquids and 178 glass types from Cargille Laboratories (USA), demonstrating that liquids span a wider range of Abbe numbers and often deviate from the normal dispersion line of glasses, exhibiting pronounced anomalous dispersion, resulting in performance comparable to conventional apochromatic systems [127].

The achromatic performance of liquid lenses fundamentally relies on multi-parameter coupled optimization. A single-lens system cannot correct chromatic aberration across the full focal range due to limited degrees of freedom in material dispersion and optical power, nor can it simultaneously suppress spherical aberration [128]. Wang et al. demonstrated that single liquid lenses cannot simultaneously correct lateral and longitudinal chromatic aberrations [129]. A two-liquid-lens system constitutes the minimal configuration capable of compensating for both chromatic and spherical aberrations. Coordinated design of multi-element or multi-cavity liquid lenses is required to achieve effective correction [126,130,131]. Wang et al. compared single- and two-liquid-lens configurations [132], revealing that the chromatic focal shift decreased from 12,620.0413 μm in the single-lens system to 273.3078 μm in the two-lens system, approximately a 46-fold improvement, providing strong validation for the achromatic design principles of liquid lenses.

In practical applications, liquid lenses have been combined with digital conical lenses to achieve achromatic, large-depth-of-focus color holographic zooming [136,137]. Chromatic aberration has also been exploited for depth sensing (Figure 13b) [134] and phase imaging (Figure 13c) [135], where the depth channel is divided into red, green, and blue sub-channels, and the liquid lens rapidly adjusts focus to capture near- and far-focused images for each channel. Christos et al. utilized a Tunable Acoustic Gradient (TAG) lens combined with a multi-color pulsed light source. Three images were captured each time using a single color to achieve color separation and correct color leakage. Then, three axially displaced images (red, green, blue) were generated for phase reconstruction. Effectively mitigating color crosstalk in conventional quantitative phase imaging and reducing color leakage to less than 3%. The phase reconstruction speed can reach up to 150 frames per second.

#### 4.4.2. Spherical Aberration

**Figure 14:**
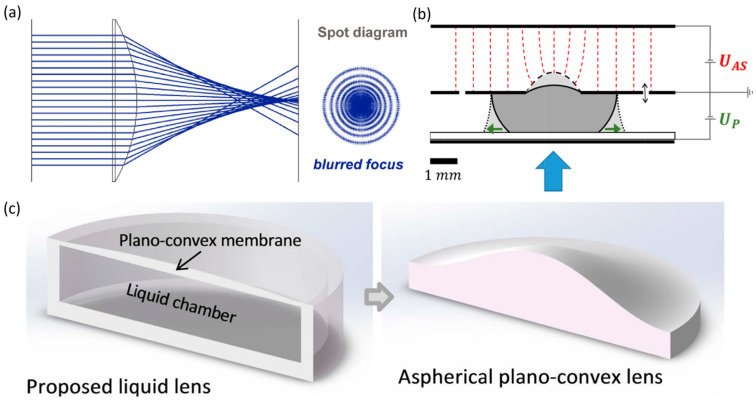
Definition of spherical aberration and approaches for aspheric implementation. (**a**) Schematic of spherical aberration in an optical path [138]. Adapted from Ref. [138] under the terms of the Creative Commons CC BY 3.0 license. (**b**) Innovation in control mechanisms: electrowetting and Maxwell stress [139]. (**c**) The elastic membrane is designed with a plano-convex cross-section, and the membrane thickness is non-uniformly distributed along the radial direction [140]. Adapted with permission from Refs. [139,140] © Optical Society of America.

For spherical lenses, the farther an incident ray is from the optical axis, the stronger its refraction, causing its focal point along the axis to be closer to the lens. Spherical aberration arises when rays passing through different regions of aperture fail to converge at a single point along the optical axis, forming a dispersed spot centered on the axis (Figure 14a). In liquid lenses, surface tension naturally shapes the meniscus into a spherical profile, inherently introducing spherical aberration. In multi-interface systems, mismatches in curvature or refractive index between interfaces can further exacerbate this aberration. Conventional solutions can be broadly categorized into multi-lens combinations and aspherical lenses. The correction logic of multi-lens combinations relies on complementary defects, similar to chromatic aberration correction. Aspherical lenses employ a variable-curvature profile, where curvature changes radially to bring rays to the same focal point, addressing spherical aberration at its source. Fabricating aspherical profiles in conventional solid lenses is costly, whereas liquid lenses can directly achieve aspherical surfaces through interface modulation, offering a natural advantage.

Correction of spherical and other aberrations is generally formulated as a multi-constraint optimization problem, involving variable control, parameter selection, and function optimization. Reichelt et al. integrated chromatic and spherical aberration correction equations into a model that treats interface curvature as the core variable under aperture radius constraints [126]. Lei et al. applied a least-squares evaluation function for optimization [131]. Zhang et al. further introduced a hybrid approach combining uniform design with deep learning [141], in which representative parameter sets (h(central membrane thickness), a(quadratic term coefficient of the convex surface contour function), b(quartic term coefficient), and c(sixth-order term coefficient)) were selected through the uniform design method to reduce simulation workload. The deep learning model then rapidly predicts the performance of all parameter combinations, facilitating the selection of globally optimal solutions for spherical and coma aberration correction.

An effective approach to correcting spherical aberration in liquid lenses is the optimization of aspherical profiles, achieved through control mechanisms [139], lens structural design [140,142], and electrode configuration [143]. Global–local cooperative control has been proposed, wherein one system regulates the overall lens shape and focal length while another fine-tunes local curvature. Mishre et al. introduced a dual electrowetting and Maxwell stress mechanism, in which high-voltage–induced Maxwell stress modifies local curvature (Figure 14b). Lens structure optimization mainly refers to integrating or designing aspherical profiles into the lens (Figure 14c). Electrode design serves as a critical approach for aspherical shape control and plays a pivotal role in the correction of complex wavefront aberrations.

#### 4.4.3. Complex Aberrations

In practical applications, aberrations rarely appear in isolation. Astigmatism primarily arises from asymmetric deformation of the liquid interface caused by uneven stresses, such as non-uniform electric fields. Other aberrations resulting from similar asymmetric deformations include coma, distortion, and higher-order non-symmetric aberrations, such as trefoil and quatrefoil. Therefore, aberrations in liquid lenses are often complex and require systematic analysis.

Complex aberration correction typically involves real-time measurement, feedback, and compensation [144,145]. For an electrically driven liquid lens, with all other electrodes held at their initial states, the voltage of a single electrode is varied, and the resulting interface profile is recorded to extract the corresponding Zernike coefficients. A vector relationship between the voltage of a single electrode and the corresponding Zernike coefficients is thus established. The vector relationships of all electrodes form an influence matrix, which describes the mapping between any combination of voltages and the resulting interface shape. In practice, wavefront aberrations are measured using wavefront sensing techniques, such as the Shack–Hartmann sensor method, interferometry, or image-based inversion. Then decomposed into a superposition of Zernike polynomials to identify the components and magnitudes of the aberrations. Using the influence matrix, the voltages required to generate conjugate Zernike modes are computed and applied, driving the liquid lens to produce a compensatory surface shape that corrects the aberrations.

A critical strategy for correcting complex aberrations in liquid lenses is precise non-spherical control through independently segmented electrodes. Zohrabi et al. simulated electrowetting devices with 4, 8, and 16 electrodes [146]. Correcting the 0° and 45°astigmatism Zernike coefficients using two 4-electrode devices increased the Strehl ratio from 0.086 to 0.92, further improved to 0.95 with an 8-electrode configuration. Using two 16-electrode devices to correct seven Zernike coefficients raised the Strehl ratio from 0.187 to 0.834. Zhao et al. developed a 32-electrode tubular tunable fluid lens, simulating the liquid–liquid interface with a combination of COMSOL finite element analysis and MATLAB analytical modeling [124]. An influence matrix was constructed, and a least-squares optimization problem was solved using an interior-point algorithm, demonstrating the feasibility of aberration correction under both sensor-based and sensorless conditions.

#### 4.4.4. Aberration Correction of Liquid Lens Arrays

**Figure 15:**
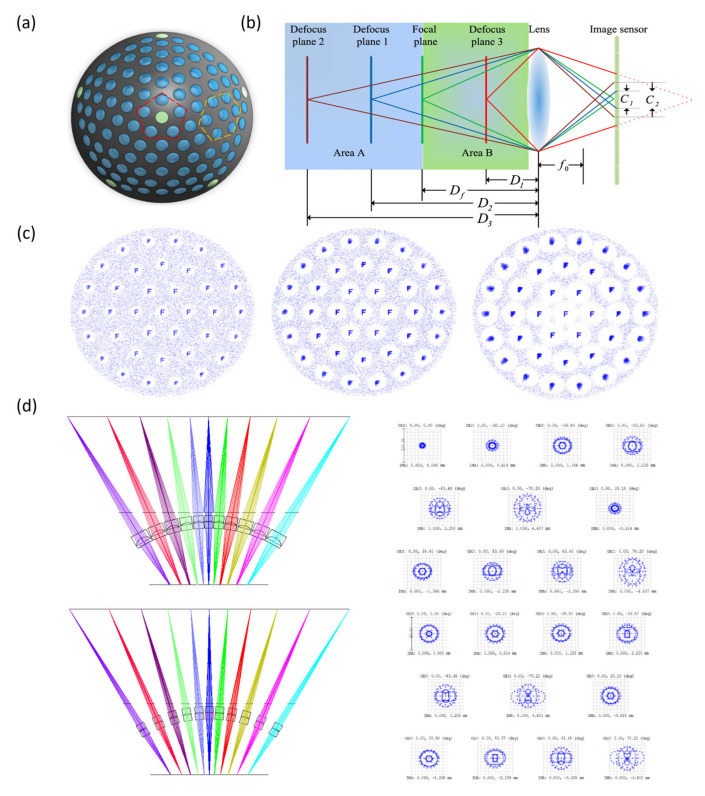
The influence of subunit diameter and size uniformity in LMLAs. (**a**) Front view of the compound eye array: pentagonal packing configuration and hexagonal packing configuration [149]. Adapted with permission from Ref. [149] © Optical Society of America. (**b**) Theoretical concept of defocus: in an ideal state, objects at the focal plane produce the sharpest images, and the spots formed by light passing through the lens are minimal. Under defocused conditions (the object moves away from the focal plane), light cannot converge precisely on the image plane, and the resulting spots gradually enlarge [154]. Adapted from Ref. [154], with permission from Taylor & Francis. (**c**) As subunit diameters increase from left to right, imaging becomes progressively blurrier, indicating more severe defocus [153]. (**d**) Comparison of spot diagrams between a non-uniform diameter array (lens diameters increase from the inner to outer rings) and a uniform diameter array: In the non-uniform array, spots at the focal plane are minimal, but as the object moves away from the focal plane, the spots enlarge significantly, indicating pronounced defocus. In contrast, the uniform array produces spots of roughly consistent size at different object distances relative to the focal plane, demonstrating improved defocus performance [153]. Adapted from Ref. [153], with permission from Opto-Electronic Engineering.

In designing LMLAs, subunit shape, center-to-center spacing, and arrangement pattern critically influence optical energy concentration and imaging quality. Polygonal subunits provide a higher effective optical area (or called fill factor) than circular ones [147]. Hexagonal layouts concentrate spot energy more effectively than square arrangements (Figure 15a). Increasing the spacing between subunit centers disperses spot energy, reducing imaging quality [148]. In compound-eye configurations, subunit spacing and arrangement also determine field-of-view overlap, affecting the overall visual coverage [149]. However, polygonal subunit apertures (such as squares) may introduce boundary-induced aberrations (quatrefoil aberrations). Kim et al. demonstrated that the boundary wall of polygonal subunits has an optimal thickness at which the lens surface closely approximates the ideal shape, minimizing wavefront error [150].

Biomimetic design of liquid lens arrays can accommodate light incident at various angles, expanding the FOV and enabling panoramic imaging. In such compound eye systems, sub-lenses in different concentric rings are prone to defocus aberrations due to variations in object distance [151,152,153], while sub-lens diameters and uniformity further influence imaging performance. Defocus introduces variations in image sharpness and spot morphology across object distances (Figure 15b). Zhao et al. demonstrated that larger sub-lens diameters exacerbate defocus, producing blurrier images (Figure 15c), and that non-uniform arrays with increasing diameters across the ring also exhibit pronounced defocus (Figure 15d).

LMLAs achieve complex aberration correction through zonal control, with each subunit acting as an independent phase element that introduces localized wavefront modifications. Zohrabi et al. optimized the curvature, tilt, and interface positions of 127 prism subunits to correct mixed aberrations, including nine Zernike coefficients, raising the Strehl ratio from 0.04 to 0.85 [155]. Zhou et al. proposed using the two liquid–liquid interfaces in a three-liquid subunit to correct wavefront tilt and curvature errors separately, and employing a two-liquid subunit to individually compensate for wavefront piston errors [148]. These results confirm the effectiveness of LMLAs in correcting complex aberrations.

## 5. Perspective and Conclusions

This work reviews recent advances in LMLAs from an application-oriented perspective, focusing on actuation mechanisms, fabrication strategies, and functional performance. Actuation approaches can be broadly classified into interface curvature modulation and refractive-index modulation. The former, represented by electrowetting and liquid–hydraulic actuation, achieves focal tuning via curvature control at liquid–liquid or liquid–solid interfaces, offering a large tunable range and rapid response, yet facing challenges in stability and optical aberrations. The latter relies primarily on liquid-crystal orientation to modulate the effective refractive index, enabling programmable wavefront shaping, while being constrained by polarization dependence, narrow tunable range, and low optical efficiency. More recently, double-emulsion droplets have been explored for Janus-type microlenses, where tuning is achieved via the internal liquid–liquid interface curvature. Compared with conventional liquid lenses, their droplet-based architecture inherently offers higher integration potential, making them promising for multifunctional integrated photonic devices.

The overall performance and design freedom of LMLAs are fundamentally constrained by the state of fabrication technologies. Current mainstream approaches can be broadly categorized into three types. Semiconductor-derived processes (e.g., soft lithography, nanoimprinting, replica molding, and reactive ion etching) leverage the maturity of wafer-scale manufacturing, offering high scalability, yet they exhibit limited flexibility for fabricating asymmetric 3D structures. Additive manufacturing techniques (e.g., two-photon polymerization, femtosecond laser writing, inkjet printing, and 3D printing) are well-suited for creating customized and complex 3D structures but remain constrained by a trade-off among precision, efficiency, and cost. It is difficult to meet the requirements for large-scale, low-cost production. Dynamic molding techniques employ external stimuli (light, electricity, or heat) to manipulate material phases or flow properties, which in turn enables programmable fabrication. Leveraging liquid-interface self-smoothing enables high-precision lens fabrication with minimal surface roughness, although scalable production remains limited by interface stability. Representative methods include droplet microfluidics, phase separation, and laser injection. From an application perspective, semiconductor-based fabrication is most suitable for large-scale production of consumer electronics, meeting the requirements for standardized optical component designs while achieving the necessary precision and optical consistency. Although additive manufacturing has limited large-scale production capability, it offers flexibility for small-batch, customized fabrication and is ideal for rapid prototyping of high-performance, customized optical devices. Dynamic molding provides unique opportunities for programmable, high-precision lens fabrication, though its scalability is still limited.

From an application-oriented perspective, the performance of LMLAs can be examined across several dimensions. Firstly, focal length tuning has evolved from unidirectional to bidirectional control. Innovations in cavity design and expanded contact-angle ranges have substantially extended zoom capabilities, with single liquid lenses achieving focal lengths from ±40 mm to infinity. Precise manipulation of liquid–liquid interfaces within a cavity enables optical zooming and variable magnification with a single element, simplifying system design. In LMLAs, this capability is inherited from individual lenses, though the arrangement, filling, and spatial layout of multiple units introduce complexity to imaging systems.

Secondly, FOV expansion is primarily achieved by steering the liquid–liquid interface to alter the optical axis or by constructing MLAs. A single liquid lens typically offers a FOV below 60°, but electrode or structural modulation can provide ±10° beam deflection, and combined with fisheye optics, scanning angles up to ±75° has been demonstrated, supporting applications such as Light detection and ranging (LiDAR) LMLAs provide more pronounced FOV extension; curved arrays inspired by insect compound eyes can reach ultrawide FOVs of over 160°. Individually tunable arrays offer additional degrees of freedom and have already been applied in adaptive high-resolution, wide-FOV imaging systems. Thirdly, DOF can be extended by hardware or computational means. Hardware approaches include multi-lens cooperation, stacked liquid-crystal arrays, or aperture integration, while computational methods fuse multi-focal images to form extended DOF. Each approach involves trade-offs between imaging clarity, complexity, and real-time performance.

Fourthly, aberration correction benefits uniquely from the tunable curvature and material flexibility of liquid lenses. Chromatic and spherical aberrations can essentially be formulated as a multi-parameter optimization problem involving interface curvature, liquid refractive index, and aperture size. In practical applications, the core strategy for correcting more complex aberrations remains the optimization of non-spherical phase to compensate wavefront errors, which is primarily achieved through multi-electrode partitioned control. However, realizing large-range tuning, aberration correction, and DOF extension simultaneously often requires multiple liquid lenses, which increases control complexity and introduces new wavefront errors. Hybrid optical systems that combine liquid and solid lenses have emerged as an effective means to balance tuning range, correction precision, and overall system stability.

Fifthly, at the array level, performance depends not only on optimizing individual lenses but also on the collective design and integration of multiple units. Each subunit acts as an independent tunable element, and the overall layout enables broader FOV, improved aberration correction, diverse functionalities (e.g., beam steering, optical switching), and enhanced applicability. Nevertheless, several challenges remain. LMLAs require precise micro- and nano-fabrication to realize complex structures while maintaining high optical performance. Electrical control of numerous independent units remains difficult due to multi-channel integration, complex addressing, and maintaining uniform responses. Advanced materials are required to ensure device reliability and longevity under demanding conditions. Structurally, conventional curved compound-eye arrays face field-matching issues when coupled with commercial flat detectors, often causing defocusing and degraded edge image quality, which necessitates optical redesign or corrective elements to achieve system-level compatibility.

Finally, LMLAs face both material- and system-level challenges. At the material level, no single liquid or membrane can simultaneously meet the ideal thermal, electrical, chemical, and mechanical requirements, which fundamentally limits actuation performance and device stability. Careful material selection can simplify structural design and reduce processing steps, while incorporating fillers with tailored optical, electrical, or magnetic properties offers a promising route to overcome intrinsic shortcomings. Progress also depends on the development of more efficient fabrication techniques that can support large-scale production of advanced soft materials. These advances are key to translating LMLAs from laboratory demonstrations to practical devices.

At the system level, strong coupling among key optical parameters—such as depth of field (DOF), numerical aperture (NA), and resolution—remains a fundamental barrier. Optimizing one parameter often degrades another; for example, extending DOF typically reduces image quality or field of view (FOV), while computational strategies for resolution recovery or DOF extension often come at the cost of real-time performance. Addressing these trade-offs requires co-optimization strategies that combine optical design with computational imaging, distributing the load between hardware and algorithms to achieve balanced improvements in clarity, efficiency, and adaptability. Specifically, these challenges manifest differently across application domains. In microscopy, cooperative operation of multiple lenses achieves large-range focusing but still suffers from limited FOV, while increasing aperture degrades image quality. Non-coaxial lens arrangements, field stitching, or compound-eye-inspired architectures may help reconcile wide FOV with high resolution. In medical endoscopy, liquid lenses enhance FOV, resolution, and structural simplicity, yet scanning speed remains insufficient for real-time imaging; Advances in lens materials, droplet size, device miniaturization, and battery life for wireless capsular endoscopes are critical. In display technologies, LC-MLAs enable 2D/3D switching but face light loss and slow response; higher-performance materials and LMLA-based replacement for mechanical MLAs hold promise for dynamic light-field modulation in emerging AR/VR applications. In industrial inspection and machine vision, response speed is paramount and depends on electrode design, dielectric layer thickness, lens diameter, and liquid viscosity, all of which can be optimized via modeling and design strategies.

Looking ahead, the advancement of novel functional materials, composite systems, and advanced fabrication strategies is steadily elevating the overall performance of liquid lenses, thereby accelerating their deployment in bionic devices, wearable platforms, and sensing technologies. A particularly promising direction lies in sensor–computation integration, where LMLA systems are seamlessly coupled with machine-learning algorithms. Such integration could establish dynamic measurement, feedback, and compensation, enabling adaptive control in real time. Beyond enhancing imaging quality and operational efficiency, this paradigm points toward intelligent optical systems capable of active perception, decision-making, and execution—an essential step toward next-generation smart photonic devices, with broad prospects in industrial automation, smart manufacturing, autonomous driving, and interactive sensing applications.

## Figures and Tables

**Table 1:** Common liquid materials in liquid lenses.

Material Name		Water-Based Conductive Liquid	Electronegative Fluid	Ionic Liquid	Propylene Carbonate (PC)	Glycerol
		NaCI *, KCI	Tetradecanedioic Acid	EMIMBF_4_, BMIMBF_4_ *, EMIMN(CN)_2_		
Surface Tension	γ (mN/m)	72.72 to 81.75	31.0	44.41 ± 0.59	-	62.5
Viscosity	η (mPa·s)	1.004 to 1.990	5.11	112.3	-	934
Boiling Point Temperature	T_b_ (°C)	100.05 to 101.80	165	290	241.6	98
Freezing/Normal Melting Point Temperature	T_f/m_ (°C)	−2.60 to −0.18	−32.4	−85	−48.8	18.2
Conductivity	κ (mS/cm)	8.2 to 222	2.4 × 10^−11^	3.53	-	-
Dielectric Constant	ε	<78.0	5.85	13.9 ± 0.8	66.14	46.53
Refractive Index	*n*	1.3332 to 1.3795	1.4369	1.4156 ± 0.0012	1.4189	1.4746
Qualitative Solubility		sl EtOH	i H_2_O; msc EtOH, eth	-	vs·H_2_O, EtOH, eth, ace, bz	msc H_2_O, EtOH; sl eth; i bz, ctc, chl
Actuation Type		Electrowetting	Electrowetting	Electrowetting Dielectrophoresis		Electrowetting Dielectrophoresis Liquid-filled type
Reference		[34,35,36]	[34,37]	[34,38,39,40,41,42]	[34]	[34]

The asterisk (*) indicates the material corresponding to the listed physical parameter. Qualitative solubility: Qualitative information on the solubility in other solvents (and in water, if quantitative data are unavailable) is given here. Note: i = in-soluble; sl = slightly soluble; s = soluble; vs. = very soluble; reac = reacts with the solvent; ace: acetone; EtOH: ethanol; eth: ethyl ether; bz: benzene; chl: chloroform; ctc carbon tetrachloride.

**Table 2:** (Continued) Common liquid materials in liquid lenses.

Material Name		Alkane	Fluorocarbon	Silicone Oil	Liquid Crystals	
		Dodecane *, Ethane, Hexane	Perfluorocarbon, Perfluorohexane *		E7 *, 5CB	E7 + 0.5 wt% TiO_2_
Surface Tension	γ (mN/m)	24.93	-	20.8	-	-
Viscosity	η (mPa·s)	1.383	-	50cst	-	-
Boiling Point Temperature	T_b_ (°C)	216.3	57.2	>300 (flash point)	TNI: 61.5	TNI: 63.5
Freezing/Normal melting Point Temperature	T_f/m_ (°C)	−9.55	−86.1	−41		
Conductivity	κ (mS/cm)	-	-	-	-	-
Dielectric Constant	ε	2.0120	-	-	∆ε: 14.1	-
Refractive index	*n*	1.4210	1.2515	1.4022	∆n: 0.216	∆n: 0.25
Qualitative Solubility		i H_2_O; vs EtOH, eth, ace, ctc, chl	i H_2_O; s eth, bz, chl	i H_2_O;	i H_2_O;	-
Actuation Type		Janus/double-emulsion droplets		Electrowetting Dielectrophoresis Liquid-filled type	electric driving	
Reference		[34]		[43]	[44]	

The asterisk (*) indicates the material corresponding to the listed physical parameter. Qualitative solubility: Qualitative information on the solubility in other solvents (and in water, if quantitative data are unavailable) is given here. Note: i = in-soluble; sl = slightly soluble; s = soluble; vs. = very soluble; reac = reacts with the solvent; ace: acetone; EtOH: ethanol; eth: ethyl ether; bz: benzene; chl: chloroform; ctc carbon tetrachloride.

**Table 3:** Common elastic membrane materials in liquid lenses.

Elastomer	Dielectric Elastomer			Liquid Crystal Elastomer	Hydrogel	
	VHB	PDMS	PU		Polyvinyl Chloride Gel (PVC)	Hydrogel Dielectric Gel
Temperature range (°C)	−10 to 90	−100 to 250	−40 to 10	−20 to 125	-	-
Dielectric constant (@ 1 Hz)	4.8	3	7	-	Pure PVC: 5–10	30 to 50
Young’s Modulus	1.0 to 3.0 MPa	0.1 to 1.0 MPa	10 to 17 MPa	kPa-Mpa range	PVC:DBA-1:9 to 1:1 10 kpa to 480 kpa	20–60 kPa
Strain (%)	Max. 120	Max. 380	100	Max. 400	Max. 76	500
Fatigue Life	>10^7^ @ 5%	>10^7^ Max 5%	-	-	>10^8^	-
Reference	[46]	[46]	[46,47]	[48,49]	[45]	[50]

Fatigue Life @ x% refers to the number of cycles a material can endure under cyclic loading at a strain amplitude of x%, representing its long-term usability.

## Data Availability

The data are available from the corresponding author upon reasonable request.
